# Follow-up study on the long-term effectiveness of the home-visiting program “ProKind”: study protocol for a randomized controlled trial

**DOI:** 10.3389/fped.2025.1606749

**Published:** 2025-10-08

**Authors:** Sören Kliem, Malte Sandner, Tilman Brand, Sebastian Fischer, Anna Lohmann, Marie Lisanne Schepan, Gabriella Conti, Dirk Baier

**Affiliations:** ^1^ Department of Social Welfare, Ernst-Abbe-Hochschule Jena, Jena, Germany; ^2^ Department of Business Administration, Georg Simon Ohm University of Applied Sciences Nuremberg, Nuremberg, Germany; ^3^ Department of Education, Training, and Employment Over the Life Course, Institut für Arbeitsmarkt- und Berufsforschung, Nürnberg, Germany; ^4^ Department of Prevention and Evaluation, Leibniz Institute for Prevention Research and Epidemiology (LG), Bremen, Germany; ^5^ Department of Health Sciences, University of Bremen, Bremen, Germany; ^6^ Department of Economics, University College London, London, United Kingdom; ^7^ Institute of Delinquency and Crime Prevention, Zurich University of Applied Sciences, Zürich, Switzerland; ^8^ Faculty of Law, University of Zurich, Zürich, Switzerland

**Keywords:** nurse family partnership program, sustained nurse home visiting programs (SNHVs), randomized controlled trial (RCT), follow-up, study protocol

## Abstract

**Background:**

The Nurse-Family Partnership (NFP) is an evidence-based home visiting program shown to improve maternal and child outcomes. Pro Kind is the first German adaptation of NFP, implemented between 2006 and 2012. While earlier evaluations demonstrated short- and medium-term benefits, no European trial has yet assessed long-term effects into adolescence.

**Objectives:**

This study protocol outlines the third phase of the Pro Kind randomized controlled trial (RCT), designed to evaluate the program’s effectiveness 14–16 years post-intervention. Primary aims are to assess adolescent and maternal outcomes related to mental health, parenting, risk behaviors, and life satisfaction, as well as potential long-term economic effects.

**Methods:**

The original RCT enrolled 755 pregnant women with psychosocial risk factors, randomly assigned to an intervention (*n* = 394) or control group (*n* = 361). The intervention comprised structured home visits from midwives or tandem teams (midwife + social worker) from pregnancy until the child’s second birthday. The 15-year follow-up combines self-report data (via online interviews and questionnaires) and administrative records on employment, social benefits, and criminal justice involvement. Discussion: This study represents the first long-term follow-up of an NFP adaptation in Europe. While U.S. trials of NFP provide evidence of the program’s effectiveness, these results cannot be generalized to European welfare contexts. This underscores the need for long-term evaluations of NFP adaptations in Europe to generate evidence that can inform policy and ensure evidence-based decision making.

## Introduction

Sustained Nurse Home Visiting programs (SNHVs) are considered one of the most thoroughly evaluated preventive approaches in Western industrialized nations to support socioeconomically disadvantaged families and to reduce health and developmental inequalities among children (1). These programs involve long-term (up to three years) repeated and regular home visits by trained family visitors (usually registered nurses, midwives, or social workers). Home visits typically begin during pregnancy and are continued postnatally. The Nurse-Family Partnership program (NFP) (2), developed in the 1970s by David Olds, is regarded as a prototypical example of SNHVs. The theoretical framework of the program is based on self-efficacy theory (3), human ecology (4), and attachment theory (5). NFP aims to improve prenatal health, strengthen parenting skills, and promote maternal life-course development in terms of education and employment. It comprises up to 64 home visits from pregnancy (preferably < 29 weeks of gestation) until the child's second birthday, conducted by specially trained and certified registered nurses. Since its development in the 1970s, NFP has been evaluated in the United States in several large-scale randomized controlled trials (RCTs).

The first RCT was conducted in the late 1970s in Elmira, a small town in the rural Chemung County in the Southern Tier region of New York State, with an initial sample of *N* = 400 mothers (Elmira Trial, 1978–1980) (6). For the subsequent comparative analysis, the NFP intervention group (IG: *n* = 116, nurse home visits from pregnancy until age 2) was compared with a composite control group (CG: *n* = 184, developmental screenings and, in some cases, transportation for prenatal appointments). The study primarily targeted young, first-time mothers with low educational attainment and low income. The home visits focused particularly on improving prenatal health (e.g., reducing smoking and substance use, promoting healthy nutrition), strengthening parenting skills (especially responsive caregiving and safe home environments), and fostering maternal self-efficacy and future planning, (e.g., continuing education, entering employment, preventing unplanned subsequent pregnancies). On average, approximately nine prenatal home visits (range: 0–16) and about 23 postnatal home visits (range: 0–59) were conducted during the first two years after birth, with a frequency of weekly visits during the first four weeks postpartum, followed by biweekly visits until the 21st month, and thereafter monthly visits [see (7)]. For the Elmira Trial, follow-up studies are now available up to 17 years after completion of the intervention [19-year follow-up (19yrs-fu)] (7).

The second RCT was conducted in Memphis, Tennessee, in an urban setting with a high poverty rate (Memphis Trial, 1990–1991) (8). The target group consisted predominantly of African American, unmarried, first-time mothers with low educational attainment and limited employment opportunities. A total of *N* = 1.139 mothers were enrolled in the study. For subsequent comparative analyses, the NFP condition (*n* = 228) was compared with a control condition (*n* = 515) similar to that used in the Elmira Trial. The program structure followed the model tested in Elmira but incorporated a stronger focus on health promotion in contexts of urban poverty. On average, approximately seven prenatal home visits (range: 0–18) and about 26 postnatal home visits (range: 0–71) were conducted during the first two years after birth (8). In addition to improving prenatal health, particular emphasis was placed on preventing substance abuse and enhancing socioeconomic prospects through education and employment planning [see (8)]. Follow-up assessments extend to child age 18 years (9). Furthermore, data on maternal and child mortality are available for a 20-year follow-up (10).

In the third trial in Denver, Colorado (Denver Trial, 1994–1995) (11), NFP was examined in a multi-ethnic urban setting. The multi-ethnic sample (46% Mexican American, 35% white non-Hispanic, 15% African American, 4% other) comprised first-time mothers with multiple psychosocial risk factors (including low income, single-parent status, and low educational attainment). The intervention structure followed the standard NFP protocol, with home visits delivered either by registered nurses (*n* = 235) or by paraprofessionals (*n* = 245). Both groups were compared with a control condition comprising *n* = 255 mothers. On average, registered nurses conducted approximately seven (range: 0–17) prenatal home visits and about 21 (range: 0–71) postnatal home visits from birth until the child's second birthday. Paraprofessionals conducted about six (range: 0–21) prenatal and approximately 17 (range: 0–78) postnatal visits (12). For the Denver Trial, follow-up studies are available up to the 9 years after the initial trial [9-year follow-up (9yrs-fu)] (13). Overall, the three RCTs demonstrate diverse and, in some cases, long-term intervention effects across different developmental domains. At the prenatal level, findings include a reduction in nicotine consumption during pregnancy [Elmira (14), Denver (11)], increased birth weight for boys [Memphis (15)], lower incidence of hypertensive pregnancy disorders [Elmira + Memphis, pooled (16)], fewer preterm births among very young mothers [14–16 years, Elmira (17)], and improved breastfeeding behavior [Elmira + Memphis (16)]. With regard to child health and developmental domains, improvements were observed in maternal parenting behavior during childhood and adolescence [Elmira: 1–2yrs-fu (14), Memphis: 6yrs-fu (15)], reduced rates of domestic violence, child abuse, and neglect [Elmira: 4–15yrs-fu (18), Memphis: 2–3yrs-fu (15, 19), Denver: 4yrs-fu, (20)], as well as lower rates of injury-related hospital visits [Elmira: 2yrs-fu (21), Memphis: 6yrs-fu, (20)]. In medium terms, improvements were also observed in attention-related abilities [Denver: 2–6yrs-fu, (13)], cognitive abilities [Elmira: 3–4yrs-fu (14); Memphis: girls: 6yrs-fu, boys: 6–12yrs-fu (15, 22); Denver: 4yrs-fu, (20)] and language skills [Denver: 2–6yrs-fu (13, 20)] in children, as well as lower prevalence rates of emotional problems and behavioral difficulties [Memphis: 6yrs-fu (15, 22), Denver: 6–9yrs-fu (13)]. Long-term follow-ups into late adolescence provide evidence of sustained effects such as lower crime rates and convictions [Elmira: 11–19yrs-fu (7), Memphis: girls: 18yrs-fu, (9)], improved academic performance [Memphis: 12–18yrs-fu (9, 23)], lower rates of adolescent substance use [Elmira: 12–15yrs-fu (16, 24), Memphis: 12yrs-fu (23)], and fewer teenage pregnancies among girls [Elmira: 19yrs-fu (7)]. Analyses from the Memphis Trial also indicate a long-term effect (20yrs-fu) of lower child mortality among NFP children compared to the control group (10). Mothers likewise benefited from the intervention in the form of fewer closely spaced pregnancies (within 24 months) and fewer abortions within 48 months postpartum (All Trials 16). Overall, NFP mothers reported lower rates of subsequent births [Elmira: 15yrs-fu (18), Memphis: 9yrs-fu (25), Denver: 4yrs-fu (20)]. In addition, NFP mothers showed higher labor force participation and reduced dependence on welfare benefits [Elmira: 4–15yrs-fu (18, 26), Memphis: 12yrs-fu (27)] as well as improvements in mental health [Memphis: 2–6yrs-fu (15)]. Furthermore, there were fewer maternal arrests [Elmira: 4–15yrs-fu (18)], fewer impairments due to alcohol and drug use [Elmira: 4–15yrs-fu (18)], and a reduced maternal mortality rate [Memphis: 20yrs-fu (10)].

In addition to the three original U.S. NFP studies (Elmira, Memphis, Denver), a recent cluster-randomized controlled trial (CRCT) was conducted to examine the scalability and implementation of NFP within the U.S. Medicaid system in South Carolina [2016–2020, *N* = 5.670 Medicaid-eligible mothers (28)]. The target group comprised first-time mothers eligible for Medicaid who often face multiple stressors such as poverty, limited access to health services, and unstable living conditions. The program structure followed the NFP standard (home visits by nurses from pregnancy until the child's second birthday; prenatal: 9 visits median, 18 mean) but was more strongly integrated into public service structures and supplemented with components to improve birth outcomes (e.g., prevention of preterm birth) and to reduce hospitalizations. The focus was on preventive health, parenting competence, and life planning, with particular attention to feasibility in large-scale state welfare-programs. The results to date (follow-up assessments are planned up to child age nine years) showed no significant effects on the primary birth outcomes [e.g., preterm birth, low birth weight, small for gestational age, perinatal mortality (28), birth intervals (1yr-fu) (29); or child mortality, severe injuries, and indicators of child abuse and neglect (2yrs-fu) (30)].

In addition, two further U.S. RCTs have been conducted on the SNHV program Minding the Baby (MtB), with partially long follow-up assessments [phase 1: 3–5yrs-fu, 2002–2005, *N* = 105 mothers at risk of adversity (31, 32); phase 2: 8yrs-fu, 2008–2011, *N* = 124 mothers at risk of adversity (33, 34)]. MtB was developed at Yale University in the early 2000s and targets first-time mothers and mothers aged 14–25 years (32, 35). Home visits usually begin during pregnancy and continue until the child's second birthday. Unlike NFP, home visits are conducted in a tandem model by advanced practice nurses and clinical social workers. The program focuses on strengthening the attachment relationship, promoting parental sensitivity and reflective functioning, and supporting the child's healthy emotional and cognitive development. The U.S. MtB studies have shown that children were more likely to have secure attachment and less likely to have disorganized attachment (31, 33) and, in infancy, exhibited fewer interaction problems (among adolescent mothers, 32). Additionally, MtB families were more likely to be fully vaccinated, young mothers were less likely to have a subsequent child after their first birth, and there were fewer child protection reports (31). In later follow-ups (3–5yrs-fu), lower levels of externalizing behavior problems (32), lower obesity rates (35), fewer general behavior problems were found. In [8yrs-fu] more frequent supportive parenting practices were found (34).

In addition to these U.S. trials, various international and high-quality evaluations (i.e., RCTs) of SNHVs have been conducted in countries with universal healthcare systems. These include international adaptations of NFP from the Netherlands [VoorZorg, 2yrs-fu, 2007–2009, *N* = 460 young [<25 years], first-time mothers with low socioeconomic status [SES] plus at least one additional risk factor (36, 37)], England [Building Blocks [BB], 7yrs-fu, 2010–2013, *N* = 1.645 teenage [<20 years] mothers with low SES (38, 39)], Germany [ProKind, 7yrs-fu, 2006–2010, *N* = 755 first-time mothers with low SES plus at least one risk factor (40–42)], and Canada (British Columbia Healthy Connections Project [BCHCP], 2yrs-fu, 2013–2016, *N* = 739 first-time mothers with low SES (43)], as well as adaptations of MtB from the UK [2yrs-fu, *N* = 148 young [<25 years] mothers (44)], and Denmark [2yrs-fu, 2018–2022, *N* = 256 young [<25 years] mothers (45)]. Furthermore, two additional RCTs are available for the Australian right@home (AUS) program [6yrs-fu, 2013–2014, *N* = 722 mothers with at least two risk factors (46, 47)] and its precursor, the Maternal Early Childhood Sustained Home-visiting (MECSH) program [2yrs-fu, 2005–2008, *N* = 208 mothers with at least one risk factor (48)]. Similar to NFP, home visits in these programs are delivered by registered nurses and extend from pregnancy until the child's second birthday but include additional modules to promote language and literacy development [see (49, 50)].

While SNHV studies conducted in universal healthcare contexts have reported some positive effects regarding birth and pregnancy outcomes [smoking: VoorZorg (51)] as well as positive medium-term outcomes (0–4yrs) for mothers [e.g., parental responsiveness: ProKind (52), MECSH (53), right@home AUS (49); parental self-efficacy: MECSH (53), ProKind (54), right@home AUS (49); breastfeeding: BB (55), VoorZorg (51), MECSH (56); postpartum health: MECSH (56), right@home AUS (49); home learning environment: ProKind (57), right@home AUS (46), VoorZorg (37)], and children [e.g., cognitive and psychomotor development: BB (55), ProKind (57); language: BB (55), right@home AUS (49); behavioral difficulties and emotional problems: MtB UK (58), VoorZorg (37)].

To date only three of these studies have provided extensive long-term (>6 yrs) evaluations. First, the BB study in England (39) linked educational, health and social administrative data up to age seven and reported modest benefits in reading (key stage 1), but no consistent effects across other educational (writing, mathematics, scientific understanding), health (e.g., use of emergency departments due to injuries or ingestion) or child protection outcomes (e.g., reports to social services and official designations as “Child in Need”). Second, the 6yrs-fu of the Australian right@home program (59) reported improvements in children's socio emotional adjustment, social competence and executive functioning, alongside higher maternal wellbeing and warmer parenting, although no significant effects were found for children's general health, school achievement, emotional abuse or maternal distress, general health or self-efficacy. Compared to these studies, the German ProKind trial provides a comparable but slightly longer observation window. At the 7yrs-fu, the program was associated with significant reductions in internalizing and externalizing child behavior, higher maternal wellbeing, and decreases in abusive and neglectful parenting [see (41, 42)].

Building on this evidence, the present paper introduces the protocol for the long-term follow-up of ProKind (approx. 15yrs-fu). This study will be the first to examine whether the intervention effects of an SNHV program in a European welfare state persist into adolescence, thereby extending the existing international evidence beyond early and middle childhood and into a developmental stage marked by heightened social, emotional, and behavioral challenges (60).

## The German adaption of NFP – the ProKind study

In Germany, the concept of early intervention (“Frühe Hilfen”) was introduced in the 1970s as part of healthcare and child welfare programs [Nationales Zentrum Frühe Hilfen (NZFH)]. Despite the development of numerous initiatives, there was a lack of comprehensive evaluation research (61). To address this gap, the Federal Ministry for Family Affairs, Senior Citizens, Women and Youth (BMFSFJ) launched the “Early Support and Social Early Warning Systems” program in 2006. These efforts led to increased attention and conceptual differentiation of early support measures in Germany, particularly regarding prevention and child protection. One such initiative is the ProKind study, a multicenter RCT adapting the NFP program for Germany (40). The intervention within the ProKind study was conducted between 2006 and 2012 in the German federal states of Bremen (a city-state in northern Germany known for its urban environment and relatively high levels of social disparities), Lower Saxony (a largely rural state with pockets of urbanization and a diverse socio-economic landscape), and Saxony (an eastern state characterized by its post-reunification socio-economic challenges and unique demographic structure).

## Sample at baseline and randomizations procedure

At the time of baseline data collection (2008–2009), women in the 12th to a maximum of the 28th gestational week were recruited based on the following inclusion criteria: (a) an ongoing pregnancy (<28 weeks), (b) at least one financial risk factor [e.g., receipt of unemployment benefits (ALG II), debt], (c) at least one additional social or personal stress factor (e.g., being underage, lack of a school diploma, personal experiences of abuse or neglect) and (d) at least basic knowledge of the German language to enable communication with the German-speaking family companions (≙ A1–A2 language level). Participants were recruited through various intermediaries, including gynecologists, midwives, youth welfare offices, psychosocial counseling centers, and employment agencies. In total, *N* = 1.157 interested pregnant women registered to participate in the project. Of these, *n* = 263 did not meet the inclusion criteria, and *n* = 139 declined participation after receiving detailed information about the project. The final sample of *N* = 755 women was randomly assigned to an intervention group (IG, *n* = 394) or a control group (CG, *n* = 361) using Efron's Biased Coin Design (62), stratified by municipality (urban vs. rural), age (≤18 vs. >18 years), and maternal nationality (German vs. non-German). The women were, on average, 21 years old at the start of the project; approximately 88% were unmarried (about 28% were single parents), and about 12% had a migration background. Only about 18% reported that the current pregnancy was unwanted. The CG did not receive any study-related interventions but had access to the regular services of the German healthcare system (*treatment as usual*, TAU). This includes the statutory right to midwifery care during pregnancy, childbirth, and the postpartum period, as enshrined in the German Midwifery Act (HebG) and the Midwifery Training and Examination Regulation (HebAPrV).

## Adaptation

Although the adaptation of the NFP program closely adhered to its original guidelines and curriculum, several key differences were implemented in the German version. A significant change was made regarding the professional background of the family companions. While the U.S. NFP program employs family nurses (specially trained pediatric nurses), the ProKind study utilized state-certified and NFP-trained midwives as family companions. This change was based on the strong integration of midwives in Germany's primary healthcare system. In Germany, midwifery is a highly respected profession providing care and support to individuals during pregnancy, childbirth, and the postpartum period. The legal foundations are regulated through the Midwifery Act (HebG) and the Midwifery Training and Examination Regulation (HebAPrV), which define the requirements for professional practice, including a state-recognized three-year training program with theoretical and practical components, a state examination, regular continuing education, and comprehensive documentation duties. Additionally, midwives must document all treatments and maintain these records for at least ten years. Furthermore, German law guarantees every woman statutory midwifery care during pregnancy, delivery, and the first twelve weeks postpartum. This statutory assistance, however, focuses exclusively on health-related aspects, is limited in duration, and does not follow a structured curriculum. All family companions in the ProKind study were required to have extensive experience working with families experiencing adversities and to demonstrate a respectful and sensitive approach to the mothers. Consistent with the NFP model, only female family companions were employed to foster trust and rapport with the participants.

Additionally, the ProKind study evaluated two different delivery models for home visits. In the first model, visits were conducted exclusively by midwives, referred to here as the Continuous Model (CM), which closely aligns with the original NFP program. In the second model, referred to as the Tandem Model (TM), the intervention was carried out by a team consisting of a midwife and a state-certified social worker (equivalent to a bachelor's degree in social work). In this model, the midwife conducted visits during pregnancy, while the social worker took over approximately two months after the birth. The conceptual rationale for the TM was twofold: (a) In Germany, prenatal and perinatal care provided by midwives is generally covered by statutory health insurance, while early childhood social support for disadvantaged families is funded through social legislation. The TM sought to align with these financing structures, and (b) the TM aimed to leverage the distinct competencies of midwives and social workers. Midwives contributed medical expertise in areas such as childbirth, infant nutrition, and early child development, while social workers provided case management skills and experience in addressing child abuse and neglect within institutional frameworks.

However, current empirical evidence indicates that the quality of the relationship between practitioner and family is a key driver of effectiveness in preventive home-visiting programs (63, 64). A central strength of the original NFP model lies in the therapeutic relationship between the nurse and the first-time mother, which is deliberately fostered through continuity, trust, and long-term engagement (9, 65). Since the TM involves a handover from midwife to social worker shortly after birth, it may potentially entail the risk of interrupting this continuity of care at a particularly sensitive developmental stage. Such a transition could make it more difficult for families to establish and maintain a stable working alliance, which is regarded as a central mechanism of program effectiveness. Continuous support by a single professional, as implemented in the CM, may therefore be more conducive to sustaining trust and engagement. Taken together, these considerations suggest that the TM could potentially entail certain structural disadvantages compared to a faithful adaptation of the original NFP design that emphasizes relational continuity as a core element of its effectiveness.

## Intervention

After enrollment in the project, participants were usually visited at home every two weeks, either exclusively by specially trained family midwives (CM) or sequentially by a tandem team of family midwives and social workers (TM). Exceptions included a weekly visit schedule in the first month of the intervention and during the 8th–12th weeks of the child's life, as well as a monthly visit schedule in the last three months of the intervention before the child's second birthday. Each home visit followed a structured procedure. In accordance with the NFP program six focus areas (domains) are addressed while working with the participants in the intervention setting. These domains are generally regarded as the most important risk and protective factors for the prevention of negative pregnancy outcomes, child abuse or neglect, developmental delay and limited economic independence. The topics of each visit were divided into six domains:

1. Maternal Health: This area was the focus during pregnancy and covered topics such as managing physical changes, birth preparation, healthy nutrition, physical activity, sleep, oral hygiene, and the consumption of nicotine, alcohol, and substances.
2. A Healthy Environment: This involved an assessment of the home environment, the implementation of safety measures to prevent accidents, securing household items, and addressing issues such as mold and secondhand smoke.
3. Personal Future Plans: The focus was on future planning (including returning to education or work, and further family planning), as well as organizing daily life and identifying personal strengths and weaknesses.
4. Maternal/Parental Role: This area took up most of the time during home visits from the birth of the child until the child's second birthday. It covered topics such as infant care, nutrition, development, parenting, and promoting positive parent-child interactions.
5. Social Networks: This addressed social support from partners, parents, and friends, the maintenance of family and friendship relationships, conflict resolution, and non-violent communication.
6. Use of Social and Health Services: Information was provided about services important for addressing immediate needs and building support networks, including prenatal check-ups, child health examinations, mother-child groups, and official appointments.

A total of *K* = 62 home visitors delivered the intervention, consisting of *k* = 37 midwives, *k* = 24 social workers, and one pediatric nurse. The average age of the home visitors was 40 years (*SD* = 7.8) at the start of the intervention. All home visitors were female and held German nationality. Each home visitor underwent approximately 16 days of in-service training, following the NFP curriculum. The NFP guidelines were translated into German, supplemented with culturally and contextually appropriate materials, and delivered by the German supervisory team. Training sessions covered program theories and their practical applications, including guidelines and specific intervention modules. While there were some profession-specific training modules (e.g., client-centered communication for midwives, feeding practices in early childhood for social workers), the curriculum was generally consistent across both staffing models. Moreover, home visitors received about one hour of clinical supervision weekly. All supervisors (*N* = 6, each with a degree in social work or psychology and additional coaching qualifications) completed a five-day training on the core principles of the program at the NFP National Office in the United States. Overall, the families received an average of 32.7 visits (*SD* = 18.6), with a range of 0–94 visits, which is slightly comparable with U.S. NFP trials ranging from approx. 28 visits (Denver) to approx. 33 visits (Memphis) [see (66)]. Furthermore Sandner (66) demonstrates that the typical distribution of visit content across pregnancy, infancy, and toddlerhood in ProKind closely matched NFP averages and recommendations, particularly in core areas such as maternal health, maternal and parental role, and life course development. Across all families, approx. 13.000 home visits were conducted within the Pro Kind project. The average length of a home visit was 82 min (*SD* = 12.4). Families in the CM received on average 32.4 visits (*SD* = 18.1), while those in the TM received 33.0 visits (*SD* = 19.4), with no statistically significant difference between the two groups. The average duration of the visits was 82.6 min (*SD* = 12.4) in the CM and 79.6 min (*SD* = 14.3) in the TM.

## Program participation

As in the original NFP program, participation in Pro Kind was voluntary. Consequently, not all mothers in the experimental group remained in the program until their child's second birthday, and therefore, not all program content could be delivered as intended. A total of *n* = 166 out of the *n* = 394 randomized mothers in the experimental group (42.2%) dropped out of the intervention prematurely. The reasons for termination can be divided into endogenous and exogenous causes: Endogenous termination causes are those deliberately or indirectly caused by the mother, such as an explicit request to terminate the program or loss of contact. Exogenous termination causes are those resulting from external circumstances, such as the child being taken into custody by the youth welfare office, the sudden death of the child, or the mother relocating to an area where further home visits could not be provided. During the first intervention phase (before the birth of the child), *n* = 52 mothers terminated the program prematurely, of which *n* = 38 (73.1%) were endogenous terminations. During the second intervention phase (after birth up to the child's first birthday), an additional *n* = 87 mothers dropped out, with *n* = 48 (55.2%) being endogenous terminations. During the last intervention phase (from the child's first to second birthday), only *n* = 27 more mothers did not complete the program as intended, with *n* = 17 (63.0%) due to endogenous termination causes. Comparing program participation between the two staffing models revealed that program participation at the end of the implementation was higher in the TM compared to the CM [TM: 47% vs. CM: 38%, *p* = .06 (67)]. Overall, the rate of 42.2% is comparable to the results in the latest US trial of the NFP reporting 44% program discontinuation [see (11)].

## Results of completed follow-up phases

ProKind has been evaluated in two follow-up phases: The first project phase (Phase 1) spanned from the 36th gestational week (GW) to the age of 36 months of the reference children (54) and was funded by the German Federal Ministry for Family Affairs, Senior Citizens, Woman and Youth [BMFSJ, funding code: IIA6-25080820V6], the Günter-Reimann-Dubbers Foundation [no funding code available], the Dürr Foundation [no funding code available], and the TUI Foundation [no funding code available]. The second project phase (7yrs-fu, phase 2) was conducted at the time of school entry (ages 6–10) of the reference children (40, 41) and was funded by the German Federal Ministry of Education and Research (BMBF funding code: 01EL1408; now: BMFTR).

**Figure 1:**
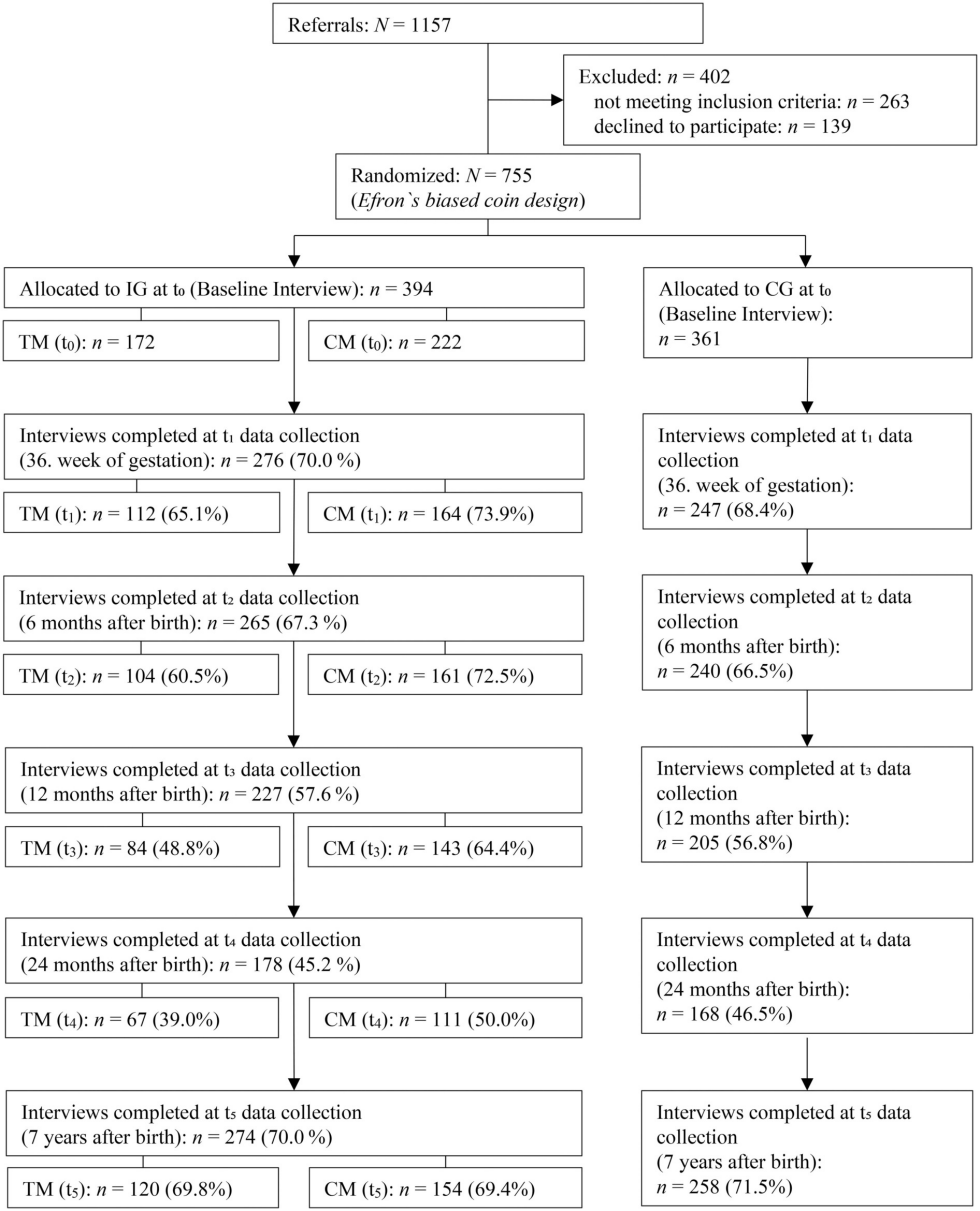
Flowchart of response rates broken down by IG and CG, with subdivision of IG into CM and TM (t0–t5). IG, intervention group; CG, control group; TM, tandem model; CM, continuous model.

The number of completed interviews at each follow-up assessment is summarized in Figure 1 by study arm (IG vs. CG) and intervention model (CM vs. TM). In the IG (*n* = 394), *n* = 276 interviews (70.0%) were completed at 36 weeks of gestation, *n* = 265 (67.3%) at child age six months, *n* = 227 (57.6%) at 12 months, and *n* = 178 (45.2%) at 24 months. In the CG (*n* = 361), *n* = 247 interviews (68.4%) were completed at 36 weeks of gestation, *n* = 240 (66.5%) at six months, *n* = 205 (56.8%) at 12 months, and *n* = 168 (46.5%) at 24 months. At the 7yrs-fu, *n* = 274 interviews (70.0%) were completed in the IG and *n* = 258 (71.5%) in the CG.

In the CM, *n* = 161 interviews (72.5%) were completed at six months, *n* = 143 (64.4%) at 12 months, *n* = 111 (50.0%) at 24 months, and *n* = 154 (69.4%) at the 7yrs-fu. In the TM, *n* = 104 interviews (60.5%) were completed at six months, 84 (48.8%) at 12 months, *n* = 67 (39.0%) at 24 months, and *n* = 120 (69.8%) at the 7yrs-fu. These figures refer solely to completed interviews and do not reflect program participation.

Sandner and Jungmann (57), based on the BSID-II, found lower rates of cognitive developmental delay among girls in the intervention group compared to the control group, even after adjustment for relevant risk factors. This advantage was evident in the first year of life and persisted in attenuated form into the second year, whereas no differences were observed for boys. A dimensional analysis of the BSID-II further indicated consistently small, though not consistently significant, effects for girls. Sierau et al. (54), and Sandner et al. (68) identified small but statistically significant differences in favor of the intervention group with respect to parental self-efficacy, perceived social support, and reduced stress. No effects were observed for parenting behavior, attachment, empathy, locus of control, life satisfaction, children's socio-emotional development, or health-related behaviors. Conti et al. (52), using micro-analytical observations, demonstrated context-dependent improvements in mother–child interactions for mothers and daughters. By contrast, no positive effects, and in some cases even negative effects, were found for boys and their mothers.

Administrative data analyses likewise indicated selective advantages. Sandner et al. (68) reported lower prescription rates of certain medications and higher utilization of dental preventive care during pregnancy, while no consistent differences emerged with regard to children's medical service use. Herrmann et al. (69), in a subsample, found more favorable maternal dental health values in the intervention group. Finally, Sandner (66) documented higher fertility rates and more favorable pregnancy outcomes among mothers in the intervention group.

Kliem and Sandner (41), in the context of the 7yrs-fu of the German Pro Kind program and based on the preregistered primary outcome domains [see (40)], demonstrated that the intervention produced significant improvements across several key outcome areas. Children in the IG showed lower rates of internalizing, externalizing, and overall behavioral problems compared to the CG, with effects being particularly pronounced among boys. Mothers in the IG also reported higher life satisfaction and reduced psychological distress. In terms of parenting practices, there was a significant reduction in abusive behavior (with boys additionally experiencing fewer instances of neglect) as well as less dysfunctional parenting behavior.

In a differentiated analysis, the effectiveness of the two implementation models of the ProKind program was examined independently. Schepan et al. (42) demonstrated that the CM proved superior in several outcome domains. It showed greater improvements in maternal mental health, maternal life satisfaction, and reductions in both dysfunctional and abusive parenting practices. In addition, the CM displayed tendencies toward advantages in reducing externalizing behavioral problems in children. By contrast, the TM was only effective in reducing children's internalizing behavioral problems. No effects were found for children's academic performance or life satisfaction. Additional analyses by Conti, Kliem & Sandner (70) indicated that only the CM was associated with a significant reduction in the 12-month prevalence of affective disorders (ICD F30–F39) among mothers and hyperkinetic disorders (ICD F90–F98) among children. Moreover, the CM was linked to lower rates of severe accidents requiring hospitalization as well as a tendency toward fewer foster care placements (71).

## The third phase of the ProKind research

This study protocol focuses on the third ProKind program phase funded by the BMBF (funding code: 01EL2013). Up to date no SNHV study conducted within a universal healthcare system has examined the long-term follow-ups of the U.S. NFP-studies (with observation periods of to 20 years). The primary goal of this research project is therefor to evaluate the long-term effectiveness of the ProKind program for adolescents aged approx. 14–17 years and their mothers (15yrs-fu; third project phase). This time point appears particularly important, as adolescence represents a critical transitional phase in which cognitive, academic, and socio-emotional competencies are consolidated that are highly relevant for the subsequent life course. During this developmental period, profound biological maturation processes (e.g., hormonal and neurobiological changes during puberty) overlap with significant cognitive and psychosocial developments, such as changes in emotion and self-regulation, self-concept, and the structure and meaning of peer relationships (60, 72). Adolescence is also considered a particularly sensitive period for the first onset of mental disorders from a developmental psychopathology perspective (73). In addition, this life stage is characterized by a marked increase in risky behaviors (e.g., substance use, sexual risk-taking) and engagement in illegal activities (74–76). These patterns are closely linked to the biological and psychosocial developmental processes of adolescence, such as heightened sensitivity to reward stimuli and increased sensation seeking, combined with still immature cognitive control capacities, as the development of the prefrontal cortex lags behind that of subcortical reward systems (77, 78). These changes interact with pre-existing risk factors from childhood, including adverse attachment experiences [e.g., (79)] and cumulative burdens of early adverse childhood experiences [ACEs; (80, 81)]. Numerous longitudinal studies have shown that ACEs (such as abuse, neglect, parental mental illness, substance-related problems, or domestic violence) are associated in a dose–response manner with an increased long-term risk of mental disorders, chronic somatic diseases, reduced social participation, and elevated mortality (82, 83). Longitudinal studies such as the Dunedin birth cohort also demonstrate that early self-control and self-regulation are linked in a dose–response relationship with later academic achievement, physical and mental health, and reduced criminality in adulthood (84). From a developmental psychology perspective, it can therefore be assumed that the early strengthening of key protective factors in early childhood (such as secure attachment, emotional and cognitive self-regulation skills, parental competence, the absence of experiences of violence, and family stability) not only reduces the likelihood of socio-emotional disorders during adolescence but also lowers the risk of problematic behaviors such as substance use, school dropout, or delinquent behavior. Long-term follow-ups of the U.S. NFP over periods of 15–20 years support this assumption, demonstrating sustained positive effects on educational attainment, psychosocial adjustment, and reduced delinquency rates (9, 85). With regard to the ProKind study, the results of the 7yrs-fu (lower rates of internalizing and externalizing behavioral problems, higher maternal life satisfaction and reduced psychological distress, reductions in abusive and neglectful parenting practices) (41, 42, 70) can, in context of existing longitudinal evidence, be interpreted as indicative of potential long-term effects. Longitudinal studies have shown that early externalizing disorders are associated with later delinquency, substance abuse, and school dropout (86–88), whereas emotional neglect and maltreatment markedly increase the risk of depression, anxiety disorders, and relationship difficulties in adolescence and adulthood (89, 90).

**Figure 2:**
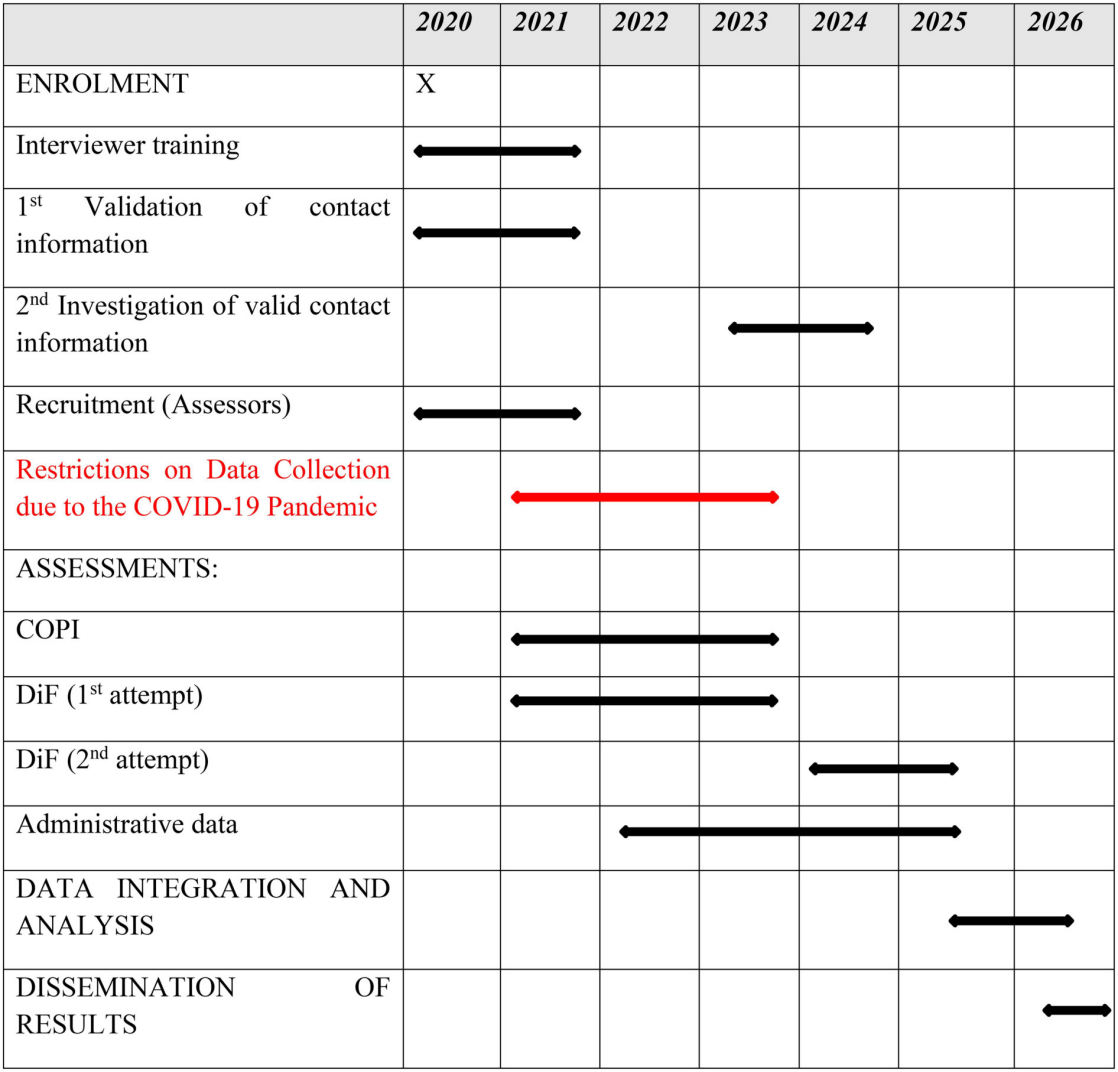
SPIRIT template of the schedule of enrolment, interventions, and assessments.

The follow-up study can be divided into six domains: (a) interviewer training, (b) collection of contact information, (c) data collection, (d) acquisition of administrative data, and (e) data analysis. The participating institutions are involved in these domains as follows: Ernst-Abbe-Hochschule – University of Applied Sciences (EAH): (a), (b), (c), (e); Institute for Employment Research in Nuremberg (IAB) of the Federal Employment Agency: (d), (e); Leibniz Institute for Prevention Research and Epidemiology (BIPS): (e). In accordance with SPIRIT guidelines (91) an overview of the ProKind follow-up schedule can be found in Figure 2. Starting in March 2020, the Covid pandemic and related containment measures caused significant disruptions to the project's feasibility and led to a substantial delay in the planned timeline. First, during the acute phase of the pandemic, data collection could not be carried out as originally planned in participants' homes, as had been done in previous phases of the project (40). This was due to the unmanageable effort required to submit hygiene protocols to local health authorities and the unacceptable health risks for all parties involved, even with a hygiene protocol in place. Second, there was a risk of treatment effect contamination, as the acute pandemic situation and the varying Covid-19 containment measures were expected to impact the variables under investigation, including life satisfaction, experience of violence, and psychological abnormalities in children (92).

The exclusion of such contamination effects was deemed essential for the meaningful calculation of treatment effects and for the successful completion of the overall project. For these reasons, it was decided, in consultation with the project sponsors, to pause the start of data collection until the risk of treatment effect distortion could be considered minimal. In addition to this necessary adjustment to external circumstances, changes were also made to the mode of assessment. To avoid being permanently dependent on the unpredictable course of the pandemic, the project consortium decided to forgo home visits and instead implement digital data collection. Accordingly, online personal interviews (COPI) were conducted by trained test administrators who were blinded to the treatment condition, and digital questionnaires (DiF) were used for data collection. This required converting all test materials into an online-administrable format, which involved additional labor-intensive steps, such as establishing a technical and digital infrastructure, creating sample videos for the target group to illustrate the new survey format, and conducting pre-tests. This adaptation necessitated a reduction in the originally planned scope of administration, resulting in the inability to administer some originally intended outcome domains. As a result, the research project experienced a significant delay and underwent several changes to the original pre-registration plan (DRKS). The study documentation is available in the German Clinical Trials Register (DRKS; ID: DRKS00025962, registration date: 08 November 2021; Last update in DRKS: 09 July 2024). The DRKS has been recognized as a WHO Primary Registry since October 2008. The protocol is reported according to the Standard Protocol Items: Recommendations for Interventional Trials (SPIRIT; Supplementary Table S1).

## Procedure and re-engagement strategy

As part of the adjusted procedure, all project staff underwent extensive training at the beginning. The training content included not only the manual-compliant administration of the psychological testing procedures used but also handling challenging interview situations (e.g., with aggressive or traumatized participants). Comprehensive quality assurance measures are being implemented, including checks on the quality of the COPI after the first and second interviews, as well as, starting from the second interview, in a rhythm of ten interviews (10% supervision rate). Detailed feedback is provided to the interviewers during these reviews.

Participants were identified through a data linkage with the German municipal registration offices (Einwohnermeldeamt, EMA), which yielded updated address information for *n* = 703 families. This linkage provided the most recent address information, thereby enabling reliable contact with participants for the purposes of the follow-up study. A project assistant first initiates personal contact by sending a letter inviting participants to update their telephone number. Upon receiving this information, the assistant contacted the participant by phone to schedule appointments for the COPI interviews (mother and children) and to provide the corresponding link for the DiF questionnaire. Participation in the COPI and the DiF is voluntary. Participants face no disadvantages if they choose not to participate. Withdrawal from the study is possible at any time, either verbally or written. If a participant withdraws consent, all identifying data are deleted and the remaining data are processed anonymously. To date, this has occurred in *n* = 17 cases, corresponding to approximately 2.3% of the total sample (*N* = 755). All participants will be informed in an appropriate and simple language about the study results. Each participant (mothers and children) receives €50 as compensation for their time invested in the COPI and DiF. The Ethics Committee of University Hospital of Jena (Registration No.: 2021-2372-Bef) approved the study design and procedure. Additionally, administrative data from various sources is utilized.

Consistent with the second phase of the ProKind study, information is collected regarding prior employment, periods of unemployment, welfare receipt, participation in qualification programs during unemployment, and employment details from the Technical Data Center (FDZ) of the IAB in Nuremberg, which is part of the German Federal Employment Agency. Participants had the option to provide self-reported data but withhold permission for researchers to access administrative data. Furthermore, the data linkage with the EMA allows access to official records on mortality of both children and mothers as well as on further births of the participating mothers and their children. Regular inquiries are made to the Federal Central Criminal Register (Bundeszentralregister, BZR). This register records criminal convictions by German courts, certain decisions by administrative authorities, notes on incapacity to be held criminally responsible, and special judicial determinations, as well as subsequent decisions and facts related to these entries. When the relevant conditions are met, foreign convictions of German nationals or individuals born or residing in Germany are also entered into the register. An anonymized report for scientific research purposes is unrestrictedly possible.

Power analyses indicated that with a one-sided test at *α* = .05 and statistical power of 1–*β* = .80, the trial is adequately powered to detect small effects. Calculations were based on the original allocation proportions of the study (*n* = 394 in the IG and *n* = 361 in the CG). Three scenarios were considered. Scenario 1 assumes administrative data with an expected re-engagement of 93% (*n* ≈ 700) which allows the detection of effects of at least ES = 0.19. Scenario 2 assumes a response rate of about 70% (*n* ≈ 530) as observed in the 7yrs-fu (41) which allows the detection of effects of at least ES = 0.22. Scenario 3 assumes a more conservative retention rate of 60% (*n* ≈ 450) which allows the detection of effects of at least ES = 0.23. According to Cohen (93), these values correspond to small effect sizes.

## Research aims and objectives

The overarching aim of the ProKind long-term follow-up study is to examine whether the medium-term program effects observed in earlier phases can be sustained or re-emerge during adolescence. The trial is designed to evaluate whether early home-visiting support contributes to improved trajectories of mental health, parenting, and overall family well-being into late adolescence. In line with the theoretical model of NFP and international evidence from RCTs of intensive home-visiting programs, three primary outcomes were defined.

First, child mental health will be assessed through standardized instruments [Child Behavior Checklist, CBCL 6/18 (94); Revised Youth Self-Report 11-18, YSR/11-18R (95)], as the prevention of emotional and behavioral disorders represents the central long-term goal of the program (10, 13, 22). Furthermore, through the repeated use of the CBCL/YSR, developmental trajectories can be consistently captured and distortions caused by switching instruments can be avoided. The procedure also provides both maternal and child reports, which allows perspectives from different informants to be incorporated in a consistent manner across developmental stages.

Second, maternal mental health will be assessed through the short form of the Depression-Anxiety-Stress Scale (DASS-21) (96) as maternal psychological well-being is a crucial determinant of parenting behavior, child outcomes, and the effectiveness of family-focused prevention programs.

Third, child maltreatment will be examined using the Conflict Tactics Scales – Parent Child (CTS-PC) (97) and the Multidimensional Neglectful Behavior Scale (MNBS) (98), given that prevention of abuse and neglect is a key target of early intervention programs and strongly associated with later adverse developmental trajectories. Third, these three main outcomes are not only theoretically central but also directly comparable with other RCTs of nurse home-visiting programs, which consistently demonstrated sustained effects in these domains [e.g., (9, 27)].

Table 1 summarizes the outcome hypotheses along with their respective operationalizations, associated informants, and survey methods. The selection of instruments and scales was guided by criteria of high psychometric quality, validity, and appropriateness. All instruments employed are particularly suited to measuring family health, living conditions, and child development. Following a multi-informant approach, various tools were used to incorporate the perspectives of both the child and the mother. A key consideration is whether the underlying research questions are (a) based on a direct outcome hypothesis of the “Pro Kind” program (primary outcome), (b) based on an outcome hypothesis but considered less likely (secondary outcome), or (c) pertain to a construct measured for further research purposes but not regarded as a primary target criterion of the intervention. Due to the volume of data collected, only the primary and secondary outcome domains are presented.

**Table 1:** Approach to evaluate the long-term effectiveness of the ProKind project.

Hypothesis	Construct	Operationalization	Primary or secondary outcome domains	Informant	Data source
Hypothesis 1: The home visits have a positive impact on the child's and mothers’ mental health.	Behavioral problems of the child emotional disorders	German version of the CBCL/6-18R/German version of the YSR/11-18R	Primary outcome	Child/Mother	COPI
	Psychological stress	German version of the DASS (target-group-specific adaptation by the authors)	Primary outcome	Mother	DiF
Hypothesis 2: The home visits reduce or prevent child maltreatment and neglect.	Psychological aggression Neglectful behavior	German version of the CTS-PC: (target-group-specific adaptation by the authors)/Scale of the German version of the MNBS	Primary outcome	Child/Mother	DiF
Hypothesis 3: The home visits have a positive impact on the life satisfaction of both the child and the mother.	General life satisfaction	Questionnaire regarding life satisfaction (FLZ) Inventory to measure the life quality of children and youths (ILK)	Secondary outcome	Child/Mother	COPI
Hypothesis 4: The home visits result in improved parenting skills (less inappropriate parenting behavior or conflicts).	Mother's dysfunctional parenting	German version of the PS (short form, target-group-specific adaptation by the authors)	Secondary outcome	Mother	COPI
	Parent-teenager conflicts	German version of the CBQ-20 (target group-oriented adaptation)			
Hypothesis 5: The home visit program reduces health related risk behaviors.	Cigarette consumption Alcohol consumption Drug consumption	Lower Saxony Survey (Niedersachsensurvey)	Secondary outcome	Child	DiF
Hypothesis 6: The home visiting program reduces the child's criminal behavior.	Juvenile delinquency Crime rates	Lower Saxony survey (Niedersachsensurvey) and admin of the BZR	Secondary outcome	Child/BZR	COPI/admin
Hypothesis 7: The home visit program has a positive impact on children's school performance.	School performance	Attended school track and recorded school grades (German, English and Maths) from the last school year.	Secondary outcome	Child/Mother	COPI
Hypothesis 8: The home visits improve life expectancy.	Mortality rate	Admin on mortality	Secondary outcome	EMA	Admin
Hypothesis 9: The home visits reduce the family's use of social benefits (SGB II, SGB III and SGB VIII).	Welfare payments	Integrated employment history provided by IAB	Secondary outcome	IAB	Admin
Hypothesis 10: The home visits influence pregnancy and births.	Mothers subsequent birth Teenager pregnancy or birth	Questionnaire on planned and realized fertility; admin record of subsequent births	Secondary outcome	Mother/Child EMA	DiF/Admin

COPI, online personal interviews; DiF, digital questionnaires; SGB II, Second Book of the Social Code; SGB III, Third Book of the Social Code; SGB VIII, Eighth Book of the Social Code; IAB, Institute of Employment Research; EMA, German municipal Registration Offices (Einwohnermeldeamt); Admin, Administrative Data; BZR, Federal German Central Criminal Register; CBCL/6-18R, Child Behavior Checklist 6-18R; YSR/11-18R, Revised Youth Self Report 11-18; DASS, Depression-Anxiety-Stress Scale; CTS – PC, Conflict Tactic Scale Parent Child; MNBS, Multidimensional Neglectful Behavior Scale-Child Report; CBQ-20, Conflict Behaviour Questionnaire; PS, parenting scale.

## Measuring instruments

### Primary outcomes

#### Mental health of children

The CBCL 6/18 assesses behavioral problems, emotional problems, somatic complaints, and social competencies of school-aged children and adolescents from the parents' perspective (94, 95). The eight problem scales can be aggregated into internalizing and externalizing disorder scales and a total score. The German manual reports adequate internal consistencies for the total problem score and the internalizing and externalizing scales, with Cronbach's *α* > .80.

The YSR/11-18R, derived from the CBCL/6-18, captures competencies and problems of adolescents aged 11–18 years, similar to the CBCL/6-18, but uses a self-report measure (94). The translated version from Döpfner and colleagues was used (95). The internal consistency of the total problem score is very high (Cronbach's *α* ≥ .93), while the internalizing and externalizing behavior scales demonstrate good reliability (Cronbach's *α* > .80).

#### Maternal distress

The short form of the DASS-21, adapted via forward-backward translation, is used to assess negative emotional states based on their frequency over the past four weeks (1 = “never” to 4 = “very often”) (96). The DASS-21 includes three dimensions: depression (e.g., dysphoria and hopelessness), anxiety (e.g., autonomic arousal and situational fear), and stress (e.g., chronic, nonspecific arousal and irritability).

#### Child maltreatment and neglect

The CTS-PC, adapted via forward-backward translation, is the parent-child version of the CTS and measures psychological and physical maltreatment, neglect, and non-violent discipline of children by parents (99). In the present studies, 31 items were used in the mothers' interviews and 16 items in the children's interviews. The CTS-PC captures the subscales Non-Violent Discipline, Psychological Aggression, Physical Assault, and Neglect.

The MNBS, whose German version was developed as part of the LIFE study at the University of Leipzig, is an instrument for assessing various forms of parental behavior related to child neglect (98). Different versions of the MNBS exist, all of which measure the extent to which the following needs of the child have been neglected: physical needs (e.g., food and clothing), emotional needs (e.g., affection and support), supervisory needs (e.g., addressing misbehavior and knowing the child's whereabouts), and cognitive needs (e.g., reading or helping with homework). The MNBS is available as a self-report measure for parents of children aged 0–15 years (Form P/PS). In the present study, eight items were presented to both mothers and children.

### Secondary outcomes

#### Dysfunctional parenting behavior

The short form of the Parenting Scale (PS) is a measure designed to assess dysfunctional parental disciplinary practices (100, 101). Based on a child behavior scenario, parents are asked to rate their own behavior between a functional and a dysfunctional approach. Two subscales—overreactivity (mothers: *α* = 0.77) and laxness (mothers: *α* = 0.73)—as well as a total score (mothers: *α* = 0.79) can be calculated.

#### Children's life satisfaction

The Inventory for Assessing the Quality of Life in Children and Adolescents (ILK) is a tool for measuring the quality of life in children and adolescents (102). The quality of life is divided into different domains, which are separately assessed in the ILK: school, family, peer relationships, and interests and leisure activities. Additionally, two health-related domains—physical health and mental health—are included. Beyond the individual domains, an overall assessment of quality of life is also provided. The internal consistencies of the quality-of-life score, based on child and adolescent self-reports, range between Cronbach's *α* = .55 and .63.

#### Mothers' life satisfaction

The Short Form of the Questionnaire for Life Satisfaction (FLZ) is designed to measure relevant aspects of life satisfaction in mothers across various life domains, such as health, financial situation, leisure time, relationship with their own children, or housing (103). In addition to assessing domain-specific life satisfaction, the FLZ allows for the estimation of general life satisfaction, calculated as the sum of seven out of the ten scales. The internal consistency for the total score is satisfactory (Cronbach's *α* = .74 for mothers). The FLZ has been normed on a representative German sample, with additional norms available for various age and occupational groups.

#### Child's consumption of alcohol, cigarettes, and Cannabis

The frequency of alcohol, cigarette, and cannabis consumption is assessed using questions from the Lower Saxony Survey 2019 (104).

#### Parent-teenager conflicts

The Conflict Behavior Questionnaire (CBQ-20; adapted via forward-backward translation) is a 20-item self-report instrument designed to assess perceived conflict and communication behavior between parents and children from the mothers' perspective (CBQ-E version). The items refer to the past two weeks and are answered with “true” (agreement) or “false” (disagreement). The results of the CBQ-20 correlate at .96 with the longer version of the CBQ, which contains 73 items (105) and has demonstrated high internal consistency (106).

#### Academic performance of children

Data referring to current school type, grade repetition, educational aspirations, current grades in German, English, and Mathematics is collected.

#### Child's criminal behavior

Criminal and violent behavior are assessed using questions from the Lower Saxony Survey 2019 (104). Criminal behavior is measured with 9 items. Example items include: “Have you ever deliberately stolen something from a shop, department store, or store?” Violent behavior is assessed with 6 items. Example items include: “Have you ever demanded money or possessions (e.g., a jacket, watch, shoes) from someone and seriously threatened violence if they did not hand them over or pay?” In addition, data from the BZR for the ProKind children's/adolescents' cohort will be gathered. These data include information on criminal convictions and relevant judicial decisions.

#### Mortality rate

Data from the EMA will be obtained to assess mortality rates.

#### Social benefit receipt

The IAB provides comprehensive data on integrated employment biographies as well as data from the SGB II performance statistics, accessed via the IAB's sampling frame (LST-S).

#### Subsequent pregnancy and desire for children

In line with previous surveys in the ProKind project and as a continuation, mothers are asked about their current family life and future family planning. This includes questions regarding contraception methods or an (already realized) additional desire for children. Administrative data further allow the tracking of how many subsequent children the mothers have had. In addition, adolescents from the ProKind cohort are asked about experiences with pregnancies and births. Administrative data further allows the identification of whether adolescents of the child cohort have already become parents.

## Data analysis

The primary and secondary analyses will be conducted on an intention-to-treat basis. We will standardize and recode all continuous outcomes such that positive values correspond to beneficial effects. The reported effect sizes (ESs) will be interpreted as group differences in standard deviations (SDs) of the CG. Confidence intervals (CIs) for the ESs will be obtained using bootstrap methods with 5,000 replications. One-sided tests (*p* < .05) will be conducted under the assumption that the intervention is not harmful.

Following the analytic strategy used in the 7yrs-fu of ProKind (41), as well as in recent evaluations of the trial (42, 71), to address missing observations from families who did not participate in the follow-up and to account for the imbalance in a few baseline risk factors (e.g., maternal aggression and mental health problems) between IG and CG, treatment effects will be estimated using augmented inverse probability weighting (AIPW) in combination with the “least absolute shrinkage and selection operator” (lasso) to select relevant control and weighting variables (107).

The AIPW technique assigns weights to observed cases based on the inverse of their probability of loss to follow-up estimated by logit functions, thereby mitigating potential selection bias caused by attrition. In a second step a linear weighted function with additional control variables is estimated. The variables used in the logit functions for the weighting and the control variables for the linear weighted function are selected by lasso, a machine learning algorithm. By weighting participants according to their inverse probability of remaining in the study, the analytic sample is reweighted to more closely resemble the original randomized population, thereby improving both internal validity and generalizability of the findings. Due to findings of the last program phase, all primary and secondary outcomes will be analyzed regarding any differential effects, while controlling for children's gender (41) and delivery model (42).

## Discussion

The aim of the study is to conduct a follow-up survey regarding the effectiveness of the German adaptation of the NFP program, approximately 15 years after the intervention ended. As part of a biopsychosocial evaluation, the long-term effectiveness of the ProKind program is being assessed at the individual level for mothers and their children. This includes examining domains previously assessed in earlier phases, such as parenting competence, life satisfaction, and instances of child abuse and neglect. Furthermore, the study explores health-related risk behaviors (e.g., smoking, alcohol, and drug use), mortality rates (administrative data), fertility rates (administrative data) and legally significant behaviors (e.g., youth delinquency by self-report, administrative Data), which are particularly impactful during adolescence - a crucial stage of development. Moreover, the study will examine the long-term fiscal impacts of the home visiting program on the welfare state. In collaboration with the IAB, data linkage with social security records is conducted to analyze information on employment histories and social benefit claims of the participants. This outcome analysis is of particular interest, as data linkage ensures a very high follow-up rate of approximately 90%.

The long-term evaluation of the ProKind sample is of critical importance for two main reasons. First, no study to date has assessed the long-term effectiveness of NFP program in the European context. Second, some of the most relevant findings related to NFP have emerged five to 20 years after the intervention concluded (24, 108). With this in mind, this randomized controlled trial (RCT) was specifically designed to enable a long-term follow-up, achieved through robust retention efforts and the inclusion of measures that predict long-term outcomes, such as the cognitive development and behavior of the child. Consequently, any successes or limitations of NFP identified in trials conducted when children are two years old may not fully reflect the program's impact, as these findings could be reevaluated or even contradicted during subsequent stages of the children's development.

## Limitations

Besides its strengths, the present study also has several limitations that should be considered when interpreting the findings. First, due to the COVID-19 pandemic and the associated infection control measures, significant adjustments had to be made to the study procedures, resulting in considerable delays to the project timeline. Given the profound significance of this study for the German social and healthcare system, this cautious approach to addressing the pandemic's impact remains essential. Contamination of the research findings by pandemic-related effects would have severely limited the interpretability of the results, rendering them unreliable and unsuitable for providing actionable recommendations for the German social and healthcare landscape. Although the research plan originally envisaged the use of standardized performance measures, these could not be implemented due to pandemic-related restrictions. Instead, the assessment of educational outcomes in the present phase relies on self-reported school grades. This method raises concerns regarding the accuracy of retrospective reports by mothers or children, which may create a risk of recall bias. Moreover, educational research consistently shows that grades are only imperfect indicators of actual academic competence, particularly when compared to standardized achievement tests. In later follow-up phases beyond the scope of the present protocol, standardized tests in reading comprehension and basic mathematics could be implemented to provide a more valid and reliable assessment of children's educational outcomes.

Second, most data will be collected via self-report, which may be affected by biases such as social desirability or recall inaccuracies. This applies in particular to psychological, social, and family-related measures. At the same time, for several important domains, administrative data is available (e.g., mortality, fertility, criminal records from the Federal Central Register, social insurance data), which provide more objective indicators and help counterbalance these potential weaknesses. Future research should aim to further expand the integration of register-based information, for example by linking educational data such as school grades or certificates, as well as child welfare data from youth welfare offices (e.g., out-of-home placements or protective measures), in order to strengthen the validity and breadth of the findings. However, such linkages remain difficult to implement in the European context due to strict data protection regulations, which substantially limit systematic access to and use of such data.

Third, the high loss to follow-up during the initial project phase, with rates of about 55% by the 24-month assessment (54), which is substantially above the commonly recommended 20% threshold for RCTs. Such high attrition rates limit the generalizability of the findings, as selective participation may bias estimates toward more resilient or resourceful families and reduce confidence in the representativeness of the intervention effects. This also complicates the interpretation of long-term outcomes, since early selective loss may systematically affect which families remain available for later follow-up assessments. In contrast, retention improved considerably in the 7yrs-fu, with response rates of about 70% in both groups (41). Importantly, in this later phase advanced statistical methods were applied, including inverse probability weighting (IPW). In this procedure, the probability of remaining in the study was modeled using baseline characteristics such as maternal age, socioeconomic status, partnership status, and mental health indicators. Participants who shared characteristics with those lost to follow-up received higher weights in the analysis, thereby reducing selection bias. IPW thus not only improves internal validity but also tackles the problem of generalizability, as it makes the analytic sample more closely resemble the original randomized population. This marks a significant methodological improvement compared to Phase I and will strengthen the robustness and validity of future findings. Nevertheless, even with such procedures, some degree of residual bias cannot be entirely ruled out in long-term evaluations.

Fourth, the widespread availability of German midwifery care in the CG represents an important contextual factor. Using health insurance data, Sandner et al. (68) demonstrated that around 70% of mothers in the CG made use of at least one statutory midwifery visit. It is therefore conceivable that this high baseline level of standard care may have attenuated the likelihood of detecting additional program effects in domains such as maternal physical health or breastfeeding outcomes.

Fifth, the study sample is not fully representative of the migrant population in Germany. The inclusion criteria required sufficient German language proficiency, which effectively excluded families with limited or no German skills. As a result, the proportion of participants with a migration background was only about half of that observed in the general population. This selective exclusion reduces the generalizability of the findings. Future adaptations of early home visiting programs should make efforts to integrate additional resources, such as interpreters, or ensure the engagement of multilingual midwives and/or social workers, in order to include families with limited host-country language skills and thereby better represent this especially vulnerable group.

Lastly, the original trial design concerns the inclusion criteria for pregnant participants, which were based on a binary classification (e.g., “female gender”). From a contemporary perspective, this approach may appear restrictive and insufficiently inclusive, as it does not reflect the diversity of gender identities and experiences of pregnancy. While such a classification reflected the dominant research practices and clinical terminology of the time, current standards emphasize the importance of inclusive and precise language in both study design and reporting (109, 110). Acknowledging this limitation is important to document potential sources of bias in participant selection and to demonstrate awareness of evolving conceptualizations of gender in health research. Future adaptations of the program and related evaluations should therefore consider gender-sensitive inclusion criteria and language that better capture the diversity of people who may benefit from such interventions.

## Implications

While studies from the United States provide robust evidence supporting the effectiveness of the NFP, these findings cannot be directly applied to European countries due to contextual differences. This is particularly evident in Germany, where comparable early childhood support programs receive substantial public funding. For example, the Federal Government invested up to €177 million in the Netzwerk Frühe Hilfen (National Center for Early Support) between 2012 and 2015. However, no long-term RCT has been carried out to assess the effectiveness of home-visiting programs within the framework of early childhood support in Germany. This gap highlights the pressing need for a rigorous evaluation to ensure evidence-based decision-making in this critical area.

It is important to note that the original ProKind trial experienced relatively high attrition during the early intervention phase, which could challenge the generalizability of the results. Nevertheless, retention improved markedly by the 7yrs-fu, with approximately 70% of participants re-engaged (41). These figures compare favorably with US NFP-trials [e.g., Elmira: 15yrs-fu (83%), 19yrs-fu (78%) and Memphis: 12yrs-fu (83%) (7, 27)]. In our design we proactively address attrition through structured re-engagement strategies that include repeated data linkage. This approach enables sustained contact with participants over extended periods and ensures the feasibility of future follow-up assessments. In addition, the use of administrative data sources such as mortality records, fertility information, social insurance data, and criminal justice registries provides an important complement to self-report measures. These data reduce the risk of bias due to selective nonresponse and thereby enhance the validity of the long-term findings.
