# Supplementary material for: Follow-up study on the long-term effectiveness of the home-visiting program “ProKind”: study protocol for a randomized controlled trial
Source: Front Pediatr. 2025 Oct 8;13:1606749. doi: 10.3389/fped.2025.1606749 (PMC12540121; doi:10.3389/fped.2025.1606749)
Supplement: Supplementary file 1 [file Datasheet1.pdf]

General Consent Form

General Consent Form

Follow-up study on the long-term effectiveness of the home visiting program ProKind based on a randomized controlled research design.

I ____
(Your Name in Block Letters)

I have been informed about the study and the survey procedure (see participant information sheet). I have read and understood all the information. If I have any questions regarding this study, I can contact prokind@eah-jena.de by email.

The analysis of my data will be conducted in a pseudonymized manner, meaning that a code number will be used instead of my name. A coding list exists that links my name to this number. This coding list is accessible only to the project staff, is stored securely with password protection, and will be deleted after the data collection is completed.

I agree with the described handling of the collected data. I am aware that I can revoke my consent to the storage of my data at any time without facing any disadvantages. Additionally, I can request the deletion of all my personally identifiable data at any time. I consent to the fully anonymized data being used for research purposes.

I have been informed that, apart from legal obligations (e.g., if my life or health is acutely and immediately endangered), no personally identifiable data from the study will be disclosed to third parties.

I understand that this research project is conducted in collaboration with other scientists. I consent to the pseudonymized data collected in this study (without mentioning my name) being shared with:

a. The sponsor of the study, the Ernst-Abbe University of Applied Sciences Jena, for scientific analysis.
b. Collaborating scientists at the Institute for Employment Research (IAB) and the Leibniz Institute for Prevention Research and Epidemiology (BIPS).

I consent to my personal data being retained for re-contact in case of continuation of this study or related follow-up studies. I understand that my data will remain pseudonymized (coding list) until the final completion of data collection and/or analysis, and only project staff will have access to it. After a maximum of 20 years, my personal data will be deleted. Until then, I can request information about my personal data and request its deletion at any time.

I consent: [ ] YES [ ] NO

Furthermore, I consent to data inquiries at the Institute for Employment Research (IAB) of the German Federal Employment Agency in Nuremberg about my stored data, linking it with survey data. The IAB data includes, for example, information on employment status and unemployment periods. My contact details may be transmitted to the IAB for this data retrieval, where they will be deleted after the successful linking. Additionally, I consent to the IAB providing my current contact details to the EAH Jena if the study continues.

I consent: [ ] YES [ ] NO

Your phone number: ____

Your address:
Name: ____
Street: ____
(Additional details, if applicable): ____
ZIP Code, City: ____

___________________________________ ____
Place, Date Your Signature
