# Supplementary material for: Follow-up study on the long-term effectiveness of the home-visiting program “ProKind”: study protocol for a randomized controlled trial
Source: Front Pediatr. 2025 Oct 8;13:1606749. doi: 10.3389/fped.2025.1606749 (PMC12540121; doi:10.3389/fped.2025.1606749)
Supplement: Supplementary file 2 [file Datasheet2.pdf]

General Consent Form

General Consent Form

Follow-up study on the long-term effectiveness of the home visiting program ProKind based on a randomized controlled research design.

I ____
(Your Name in Block Letters)

I have been informed about the study and the survey procedure (see participant information sheet). I have read and understood all the information. If I have any questions regarding this study, I can contact prokind@eah-jena.de by email.

The analysis of my data will be conducted in a pseudonymized manner, meaning that a code number will be used instead of my name. A coding list exists that links my name to this number. This coding list is accessible only to the project staff, is stored securely with password protection, and will be deleted after the data collection is completed.

I agree with the described handling of the collected data. I am aware that I can revoke my consent to the storage of my data at any time without facing any disadvantages. Additionally, I can request the deletion of all my personally identifiable data at any time. I consent to the fully anonymized data being used for research purposes.

I have been informed that, apart from legal obligations (e.g., if my life or health is acutely and immediately endangered), no personally identifiable data from the study will be disclosed to third parties.

I understand that this research project is conducted in collaboration with other scientists. I consent to the pseudonymized data collected in this study (without mentioning my name) being shared with:

a. The sponsor of the study, the Ernst-Abbe University of Applied Sciences Jena, for scientific analysis.
b. Collaborating scientists at the Institute for Employment Research (IAB) and the Leibniz Institute for Prevention Research and Epidemiology (BIPS).

Consent for administrative data retrieval:

I agree that, as in the first and second project phases, requests will be made to health insurance companies and medical associations to determine the use of health services for myself and my child. Additionally, diagnostic data based on the International Statistical Classification of Diseases and Related Health Problems (ICD) will be provided.

I consent: [ ] YES [ ] NO

I also agree that data stored at the Institute for Employment Research (IAB) regarding my employment history and unemployment phases may be retrieved. My contact details may be transmitted to the IAB for this purpose and will be deleted after successful linkage. Additionally, I agree that the IAB may provide my current contact details to EAH Jena if the study continues.

I consent: [ ] YES [ ] NO

Consent for child's participation:

I allow my child to participate in the survey, which includes age-appropriate questions and tests. I can review all questionnaires. My child's answers will remain anonymous.

I consent: [ ] YES [ ] NO

Feedback on clinically relevant findings:

If psychological abnormalities, partial performance, or attention disorders are identified in you or your child during the study, we will inform you about them. You are free to seek further evaluation and, if necessary, support services. If desired, we can provide you with additional information materials.

I consent to my personal data being used for re-contact if this study continues or if follow-up studies are conducted. I understand that my data will remain pseudonymized (coding list) until the final completion of data collection and/or analysis, and only project staff will have access to it. My personal data will be deleted after a maximum of 20 years. Until then, I can request information about my personal data and request its deletion at any time.

Your phone number: ____

Your address:

Name: ____
Street: ____
(Additional details, if applicable): ____
ZIP Code, City: ____

____________________________________ ____

Place, Date Your Signature
