# Supplementary material for: Follow-up study on the long-term effectiveness of the home-visiting program “ProKind”: study protocol for a randomized controlled trial
Source: Front Pediatr. 2025 Oct 8;13:1606749. doi: 10.3389/fped.2025.1606749 (PMC12540121; doi:10.3389/fped.2025.1606749)
Supplement: Supplementary file 3 [file Datasheet3.pdf]

Allgemeine Einverständniserklärung

Folgeuntersuchung zur langfristigen Wirksamkeit des Hausbesuchsprogramms *ProKind* anhand eines randomisierten kontrollierten Forschungsdesigns

Ich ____

(Ihr Name in Blockschrift)

bin über die Studie und den Ablauf der Befragung (siehe Teilnehmerinnen-Information) aufgeklärt worden. Ich habe alle Informationen vollständig gelesen und verstanden. Sofern ich Fragen zu dieser Studie habe, kann ich diese per Mail an prokind@eah-jena.de stellen. Die Auswertung meiner Daten erfolgt pseudonymisiert, d. h. unter Verwendung einer Nummer und ohne Angabe meines Namens. Es existiert eine Kodierliste, die meinen Namen mit dieser Nummer verbindet. Die Kodierliste ist nur den Projektmitarbeiter:innen zugänglich; sie ist passwortgeschützt gespeichert und wird nach Abschluss der Datenerhebung vernichtet. Mit der beschriebenen Handhabung der erhobenen Daten bin ich einverstanden. Mir ist bekannt, dass ich mein Einverständnis zur Aufbewahrung bzw. Speicherung meiner Daten widerrufen kann, ohne dass mir daraus Nachteile entstehen. Ich bin darüber informiert worden, dass ich jederzeit eine Löschung aller meiner mich identifizierenden Daten verlangen kann. Ich bin damit einverstanden, dass die vollständig anonymisierten Daten zu Forschungszwecken weiterverwendet werden können. Ich bin darüber informiert worden, dass außerhalb gesetzlicher Verpflichtungen (z.B. wenn mein Leben oder meine Gesundheit akut und unmittelbar gefährdet ist) keine personen-identifizierenden Daten der Untersuchungen an Dritte übermittelt werden dürfen. Ich weiß, dass es sich bei dem Forschungsprojekt um eine Studie handelt, die in Kooperation mit anderen Wissenschaftler:innen durchgeführt wird. Ich erkläre mich damit einverstanden, dass im Rahmen dieser Studie erhobene Daten pseudonymisiert, also ohne Nennung meines Namens, weitergegeben werden an:

a. den Auftraggeber der Studie, die Ernst-Abbe-Hochschule Jena, zur wissenschaftlichen Auswertung;
b. kooperierende Wissenschaftler:innen, dem Institut für Arbeitsmarkt und Berufsforschung (IAB) und dem Leibniz-Institut für Präventionsforschung und Epidemiologie (BIPS).

Einwilligungserklärung für administrative Datenabrufe

Ich bin damit einverstanden, dass wie in der ersten und zweiten Projektphase Anfragen an die Krankenversicherungen und kassenärztlichen Vereinigungen gestellt werden, um die Inanspruchnahme von Gesundheitsleistungen für mich und mein Kind zu ermitteln. Darüber hinaus werden hierbei auch Diagnosen nach der Internationalen Statistischen Klassifikation der Krankheiten und verwandter Gesundheitsprobleme zur Verfügung gestellt.

Ich bin damit einverstanden: O JA O NEIN

Außerdem bin ich damit einverstanden, dass Daten abgefragt werden, die beim Institut für Arbeitsmarkt- und Berufsforschung (IAB) der Bundesagentur für Arbeit in Nürnberg über mich vorliegen. Dabei handelt es sich zum Beispiel um Informationen zu Beschäftigungsverhältnissen und Phasen der Arbeitslosigkeit. Zur Abfrage dieser Daten dürfen meine Kontaktdaten an das IAB übermittelt werden, wo sie im Anschluss an die erfolgreiche Verknüpfung gelöscht werden. Zudem bin ich damit einverstanden, dass das IAB bei einer Weiterführung der Studie meine aktuellen Kontaktdaten der EAH Jena zur Verfügung stellt.

Ich bin damit einverstanden: O JA O NEIN

Einwilligungserklärung für Teilnahme des Kindes

Ich gestatte, dass mein Kind an der Befragung teilnimmt, in welcher altersentsprechende Fragen und Tests durchgeführt werden. Alle Fragebögen können von mir eingesehen werden. Die Antworten meines Kindes bleiben anonym.

Ich bin damit einverstanden: O JA O NEIN

Rückmeldung von Ergebnissen zu behandlungsbedürftigen Auffälligkeiten

Sofern im Rahmen der Untersuchung Hinweise auf psychische Auffälligkeiten Ihrerseits oder psychische Auffälligkeiten, Teilleistungs- und Aufmerksamkeitsstörungen Ihres Kindes vorgefunden werden, werden wir Ihnen diese mitteilen. Ihnen steht es dann frei, diese Auffälligkeiten weiter abklären zu lassen und gegebenenfalls Hilfsangebote in Anspruch zu nehmen. Sofern Sie dies wünschen, können wir Ihnen in diesem Falle zusätzliche Informationsmaterialien zur Verfügung stellen.

Ich gebe mein Einverständnis, dass im Falle einer Fortführung dieser Studie oder von Anschlussstudien meine personenbezogenen Daten für eine erneute Kontaktaufnahme weiterverwendet werden dürfen. Ich bin darüber informiert, dass meine Daten bis zum endgültigen Abschluss der Datenerhebung und/oder Auswertung weiterhin in pseudonymisierter Form (Kodierliste) vorliegen und nur Projektmitarbeitende darauf Zugriff haben. Nach spätestens 20 Jahren werden meine personenbezogenen Daten gelöscht. Bis dahin kann ich jederzeit Auskunft über meine personenbezogenen Daten erhalten und die Löschung meiner Daten verlangen.

Ihre Handynummer: ____

Ihre Adresse:

Name: ____

Straße: ____

(evtl. Zusatz): ____

PLZ, Ort: ____

__________________________________ __________________________________

Ort, Datum Ihre Unterschrift
