# Supplementary material for: Follow-up study on the long-term effectiveness of the home-visiting program “ProKind”: study protocol for a randomized controlled trial
Source: Front Pediatr. 2025 Oct 8;13:1606749. doi: 10.3389/fped.2025.1606749 (PMC12540121; doi:10.3389/fped.2025.1606749)
Supplement: Supplementary file 4 [file Datasheet4.pdf]

Allgemeine Einverständniserklärung

Folgeuntersuchung zur langfristigen Wirksamkeit des Hausbesuchsprogramms Pro Kind anhand eines randomisierten kontrollierten Forschungsdesigns

Ich ____________________________________

(Dein Name in Blockschrift)

bin über die Studie und den Ablauf (siehe Informationsblatt der Befragung) aufgeklärt worden. Ich habe alle Informationen vollständig gelesen und verstanden. Sofern ich Fragen zu dieser Studie habe, kann ich diese per Mail an prokind@eah-jena.de stellen. Die Auswertung meiner Daten erfolgt pseudonymisiert, d. h. unter Verwendung einer Nummer und ohne Angabe meines Namens. Es existiert eine Kodierliste, die meinen Namen mit dieser Nummer verbindet. Die Kodierliste ist nur den Projektmitarbeiter:innen zugänglich; sie ist passwortgeschützt gespeichert und wird nach Abschluss der Datenerhebung vernichtet. Mit der beschriebenen Handhabung der erhobenen Daten bin ich einverstanden. Mir ist bekannt, dass ich mein Einverständnis zur Aufbewahrung bzw. Speicherung meiner Daten widerrufen kann, ohne dass mir daraus Nachteile entstehen. Ich bin darüber informiert worden, dass ich jederzeit eine Löschung aller meiner mich identifizierenden Daten verlangen kann. Ich bin damit einverstanden, dass die vollständig anonymisierten Daten zu Forschungszwecken weiterverwendet werden können. Ich bin darüber informiert worden, dass außerhalb gesetzlicher Verpflichtungen (z.B. wenn mein Leben oder meine Gesundheit akut und unmittelbar gefährdet ist) keine personen-identifizierenden Daten der Untersuchungen an Dritte übermittelt werden dürfen. Ich weiß, dass es sich bei dem Forschungsprojekt um eine Studie handelt, die in Kooperation mit anderen Wissenschaftler:innen durchgeführt wird. Ich erkläre mich damit einverstanden, dass im Rahmen dieser Studie erhobene Daten pseudonymisiert, also ohne Nennung meines Namens, weitergegeben werden an:

a. den Auftraggeber der Studie, die Ernst-Abbe-Hochschule Jena, zur wissenschaftlichen Auswertung;
b. kooperierende Wissenschaftler:innen, dem Institut für Arbeitsmarkt und Berufsforschung (IAB) und dem Leibniz-Institut für Präventionsforschung und Epidemiologie (BIPS).

Ich gebe mein Einverständnis, dass im Falle einer Fortführung dieser Studie oder von Anschlussstudien meine personenbezogenen Daten für eine erneute Kontaktaufnahme weiterverwendet werden dürfen. Ich bin darüber informiert, dass meine Daten bis zum endgültigen Abschluss der Datenerhebung und/oder Auswertung weiterhin in pseudonymisierter Form (Kodierliste) vorliegen und nur Projektmitarbeitende darauf Zugriff haben. Nach spätestens 20 Jahren werden meine personenbezogenen Daten gelöscht. Bis dahin kann ich jederzeit Auskunft über meine personenbezogenen Daten erhalten und die Löschung meiner Daten verlangen.

Ich bin damit einverstanden: O JA O NEIN

Außerdem bin ich damit einverstanden, dass Daten abgefragt werden, die beim Institut für Arbeitsmarkt- und Berufsforschung (IAB) der Bundesagentur für Arbeit in Nürnberg über mich vorliegen und diese mit den Befragungsdaten verknüpft werden. Bei den Daten des IAB handelt es sich zum Beispiel um Informationen zu Beschäftigungsverhältnissen und Phasen der Arbeitslosigkeit. Zur Abfrage dieser Daten dürfen meine Kontaktdaten an das IAB übermittelt werden, wo sie im Anschluss an die erfolgreiche Verknüpfung gelöscht werden. Zudem bin ich damit einverstanden, dass das IAB bei einer Weiterführung der Studie meine aktuellen Kontaktdaten der EAH Jena zur Verfügung stellt.

Ich bin damit einverstanden: O JA O NEIN

Deine Handynummer: ______________________________
(um später die Gutscheine zu erhalten)

Deine Adresse:

Name: ______________________________

Straße: ______________________________

(evtl. Zusatz): ______________________________

PLZ, Ort: ______________________________

______________________________ ______________________________
Ort, Datum Deine Unterschrift
